# Prevalence of respectful maternity care and associated factors among Somali refugee women following facility-based childbirth in Melkadida refugee camps, Ethiopia: a mixed-methods study

**DOI:** 10.1186/s12978-026-02435-5

**Published:** 2026-08-12

**Authors:** Kader Bare, Adiam Nega, Zeytu Gashaw Asfaw

**Affiliations:** 1 Department of Epidemiology and Biostatistics, School of Public Health, Addis Ababa University, Addis Ababa, Ethiopia; 2 Department of Health Systems Management and Health Policy, School of Public Health, Addis Ababa University, Addis Ababa, Ethiopia

**Keywords:** Respectful maternity care, Disrespect and Abuse, Childbirth, Melkadida refugee camps

## Abstract

**Background:**

Respectful maternity care (RMC) is a fundamental human right that ensures women’s dignity, privacy, and autonomy during childbirth. In humanitarian settings, however, RMC is often neglected. Despite Ethiopia hosting one of Africa's largest refugee populations, research on RMC in refugee camps, particularly in the Melkadida camps, remains limited.

**Aim:**

This study aimed to estimate the prevalence of respectful maternity care and examine factors associated with receiving respectful maternity care among Somali refugee women following facility-based childbirth in Melkadida refugee camps, Ethiopia.

**Methods:**

A convergent mixed-methods study was conducted from March 1 to March 30, 2025. Quantitative data were collected from 417 women using a structured questionnaire, while qualitative data were gathered through in-depth interviews with 16 participants. Data were analyzed using SPSS for quantitative analysis and open code software for thematic analysis of qualitative data.

**Results:**

The study had a 98.6% response rate, with 18.7% of participants reporting respectful maternity care (95% CI: 15%–24.4%). Factors positively associated with RMC included being aged 25–29 years (AOR = 3.46), attending four or more antenatal care visits (AOR = 3.77), daytime delivery (AOR = 2.20), staying less than 12 h post-delivery (AOR = 3.82), and having male providers (AOR = 3.20). Conversely, poor facility maintenance (AOR = 0.09) and moderate maintenance (AOR = 0.40) were negatively associated with RMC. Key barriers to RMC included poor infrastructure, staff shortages, insufficient training, and a lack of accountability.

**Conclusion:**

Respectful maternity care is critically low in Melkadida refugee camps. To improve RMC, it is essential to prioritize infrastructure upgrades, ensure resource availability, enhance staff training, and implement monitoring and accountability measures.

## Introduction

Respectful maternity care (RMC) is an organized care provided for all women that upholds their dignity, privacy, and confidentiality, while protecting them from harm and abuse, enables informed choices and receives continuous emotional support throughout labor and delivery [1]. RMC is grounded in a human rights-based approach to maternal health and recognizes childbirth as a critical moment requiring person-centered and compassionate care. In contrast, disrespect and abuse (D&A) refers to any form of harsh treatment or uncaring behavior towards a woman during labor and delivery including non-consented care, non-confidential care, physical abuse, non-dignified care, abandonment, detention in health facilities, and discrimination care based on personal attributes [2].

Globally by 2020, an estimated 287,000 women worldwide died due to preventable pregnancy and childbirth-related complications, 94% of which occurred in low- and middle-income countries (LMICs). In Sub-Saharan Africa (SSA), 70% of these maternal deaths were recorded [3]. These crises are exacerbated in humanitarian settings where health systems are often disrupted and access to quality care is limited putting women in such contexts at particularly higher risk of maternal morbidity and mortality [4]. In fact, maternal mortality rates in humanitarian settings are more than double the global average [5]. Women living in protracted displacement settings often face compounded vulnerabilities due to fragile health systems, resource scarcity, and social marginalization, which further compromise the quality of maternity care they receive.

Reducing maternal mortality is one of the global agendas, under the Sustainable Development Goals (SDGs), which aims to reduce the global maternal mortality ratio (MMR) to fewer than 70 per 100,000 live births by 2030 [6]. Achieving this goal requires not merely increased access to skilled birth attendance (SBA), but also a focus on the quality of care. In this regard, World Health Organization (WHO) emphasizes RMC as a key strategy to improve maternal and neonatal health outcomes, promote healthcare utilization, and build trust between women and the health system [7]. In alignment with these initiatives, the Ethiopian Ministry of Health has implemented RMC as one of the national agendas within the Ethiopian Health Sector Transformation Plan (EHSTP) to improve the quality and equity of maternal healthcare [8]. Despite these efforts, many women in LMICs continue to experience D&A during facility-based childbirth, including physical abuse, verbal abuse, abandonment, and detention in facility for inability to pay [9]. Such mistreatment during labor and childbirth discourages women from seeking facility-based services, even when they realize the potential health benefits of giving birth in the health facilities due to previous disrespectful experiences [7, 10]. This erosion of trust in health systems undermines progress toward universal health coverage and equitable maternal health outcomes.

Studies in LMICs reported high rates of D&A during facility-based childbirth: in India 99.1%, Mozambique 80%, Ethiopia 98.9%, and Nigeria 30.1%. Type of health facility, maternal education level, mode and time of delivery and socioeconomic status were some associated factors with RMC [11–14]. These challenges are further intensified in refugee settings, where women face heightened vulnerabilities due to displacement, limited rights, economic hardship, substandard living conditions, and inadequate access to healthcare. In addition, refugee women are at a significantly higher risk of sexual violence, unwanted pregnancies, and unsafe abortions [15–17]. These compounded risks, along with poor health infrastructure, limited resources, and a shortage of trained healthcare providers, severely undermine the provision of respectful maternity care (RMC) in these settings [15, 18, 19]. In such environments, RMC is often deprioritized, as the primary focus tends to be on life-saving emergency interventions, such as emergency obstetric and newborn care (EmONC) [20]. As a result, the relational and dignity-centered components of care may receive limited attention despite their importance for maternal well-being and service utilization.

While RMC is critically important, especially in contexts where women face multiple vulnerabilities, much of the existing research has focused on non-humanitarian settings [21, 22]. Evidence from refugee and humanitarian contexts remains scarce and underexplored. Notably, in Ethiopia, research on RMC in refugee settings is sparse, with no published studies assessing its status among refugee populations. This represents a significant knowledge gap, particularly considering the unique socio-cultural and structural challenges faced by Somali refugee women residing in the Melkadida camps. Therefore, the present study aimed to estimate the prevalence of respectful maternity care and examine factors associated with receiving respectful maternity care among Somali refugee women following facility-based childbirth in Melkadida refugee camps, Ethiopia.

## Methods

### Study design, period, and setting

A facility-based convergent mixed-methods study was conducted from March 1 to March 30, 2025, across five health centers within the Melkadida refugee camps, Ethiopia. Melkadida refugee is the second-largest refugee operation in Ethiopia and consists of five refugee camps: Bokolmayo, Melkadida, Kobe, Hilaweyn, and Buramino, located in the Somali Regional State, Liban Zone, approximately 1,000 km from Addis Ababa, the capital of Ethiopia, and 600 km from Jigjiga. These camps host more than 214,000 refugees, with more than 70% being children and women [23].

A mixed-methods design was intentionally selected to generate both measurable epidemiological evidence and in-depth contextual understanding. The quantitative component aimed to quantify the prevalence of RMC and identify statistical associations between demographic, healthcare access, and facility-related factors. This component provides objective estimates of magnitude and determinants of RMC within the refugee camp context. The qualitative component, on the other hand, aimed to capture the personal experiences, perceptions, and barriers to RMC from the perspective of Somali refugee women. Through in-depth interviews, this qualitative approach provided deeper insights into the contextual challenges and subjective experiences that could not be fully captured through quantitative measures alone. By integrating both components, the study offers a more comprehensive and contextually grounded understanding of respectful maternity care, linking statistical findings with women’s lived realities in humanitarian settings.

### Population and sampling

#### Source of population

For the quantitative component, all refugee women who gave birth at the refugee health centers in the Melkadida refugee camp were included. For the qualitative component, healthcare providers, community leaders, and refugee women who gave birth at these health centers were included.

#### Study population

For the quantitative component, all selected refugee women who gave birth at the refugee health centers in the Melkadida refugee camp were included. For the qualitative component, purposively selected healthcare providers, community leaders, and women who gave birth at the refugee health centers in the Melkadida refugee camp were included.

### Inclusion and exclusion criteria

#### Inclusion criteria

For the quantitative study, all Somali refugee women who gave birth within the last six months preceding the study period and visited the health centers for postnatal care, child vaccination, or other services were eligible for inclusion. For the qualitative study, the inclusion criteria were health workers with at least one year of experience, active community leaders, and women who were not involved in the quantitative survey.

#### Exclusion criteria

For the quantitative study, women who gave birth outside the refugee health centers or were critically ill were excluded. In the qualitative study, health workers with less than one year of experience, inactive community leaders, and women who had already participated in the quantitative survey were excluded.

### Sample size calculation and sampling procedure

#### Sample size calculation

The sample size for the quantitative component was determined using a single population proportion formula, assuming prevalence to be 50% (due to lack of previous studies in refugee settings) at a 95% confidence interval, with a margin of error (confidence limit) of 5%, and a 10% non-response rate.

$$n=\frac{{(z\upalpha /2)}^{2}.pq}{{d}^{2}}$$

where:

*n=* sample size.

Z(α/2) = the value of normal distribution, representing a confidence level of 95% with a value of 1.96.

*p=* anticipated proportion of RMC during childbirth, and 50% was taken to increase the sample size.

*d* = Margin of error 5%.

$$n=\frac{{\left(1.96\right)}^{2}.0.5(1-0.5)}{{(0.05)}^{2}}$$

With the above inputs, the minimum sample size needed for the study was 384.16. Assuming a 10% non-response rate, the final sample size was 423. For the qualitative component, sample size was determined based on information saturation, which was reached after sixteen participants including five HCPs, five community leaders, and six women.

#### Sampling process and recruitment

The total sample size for the quantitative study was allocated proportionally based on the number of facility deliveries recorded at each refugee health center within the six months prior to the study period (March 2025). For each health center, systematic random sampling was used to select participants. The sampling interval for each health center was calculated by dividing the total number of eligible women by the sample size determined for that health center. A random starting point between 1 and 4 was selected using a lottery method among the first four women (two were randomly chosen), after which every 4th woman was selected until the target sample size was reached. For the qualitative study, purposive sampling was employed to select 16 participants, including health workers, active community leaders, and women who were not part of the quantitative survey, to ensure a diverse range of perspectives. Data collection and analysis were conducted iteratively, with emerging themes reviewed after each set of interviews. Interviews continued until consecutive discussions yielded no new codes, patterns, or information, indicating that saturation had been reached. Information saturation, defined as the point at which additional interviews no longer generate new themes or insights, guided the sample size.

In addition to women, the qualitative component included healthcare providers and community leaders to capture multiple perspectives on respectful maternity care. These groups were included because they play a key role in shaping, delivering, and supporting maternal care services. Understanding their perspectives provides insight into how they perceive challenges and facilitators to respectful care and helps identify system-level and community-level factors that influence women’s experiences. Including these participants allows the study to not only explore women’s lived experiences but also understand how those involved in providing and supporting care recognize and address issues related to respectful maternity care.

#### Recruitment process

Eligible women for the quantitative study were identified through health center records of recent deliveries and were approached for participation during their postnatal visits. For the qualitative study, women, health workers, and community leaders were approached based on their roles and experiences, with invitations extended by the study team or through local leaders.

#### Participation rate

A total of 417 women participated in the quantitative study, resulting in a response rate of 98.6%. For the qualitative study, 16 participants were interviewed, based on the criterion of reaching information saturation.

### Operational definition

Respectful maternity care (RMC) refers to care provided to all women during labor and childbirth that upholds women’s dignity, privacy, and rights [1]. In this study, RMC was measured using 15 verification items grouped into four dimensions: friendly care (7 items assessing kindness, empathy, respectful communication, and being addressed by name), abuse-free care (3 items assessing the absence of physical or verbal abuse), timely care (3 items assessing prompt service, absence of unnecessary delays, and allowance of cultural practices), and discrimination-free care (2 items assessing the absence of mistreatment based on personal attributes). Each item had two possible responses (Yes/No) and was coded dichotomously (Yes = 1, No = 0). For each dimension, a woman was considered to have received that component of care if she responded “Yes” to all respective items. An overall RMC score was computed by summing the 15 items. A woman was classified as having received RMC only if she answered “Yes” to all items (total score = 15). Conversely, if she responded “No” to at least one item (total score < 15), she was classified as having experienced disrespect and abuse [24–27].

### Data collection procedures and tools

Quantitative data were collected using a structured and pre-tested questionnaire adapted from a validated tool on respectful maternity care (RMC) in the Ethiopian context. The tool consists of four domains with 15 verification items, which demonstrated good internal consistency (Cronbach’s alpha (α) = 0.857) and adequate reliability (α = 0.845) [24]. The questionnaire also included sections for socio-demographic, obstetric, healthcare provider-related, and facility infrastructure factors, developed based on a review of topic-related literature [25, 28–30]. The questionnaire was developed in English and then translated into Somali, ensuring linguistic appropriateness for the participants. The translation process followed a back-translation method to ensure accuracy: the Somali version was translated back into English by a different translator.

To address literacy considerations, the questionnaire was administered with support from trained enumerators. Participants filled in the questionnaire with the assistance of enumerators, who were fluent in Somali and were trained to guide the participants through the questions. This support was particularly important for women with low literacy levels, ensuring they could provide accurate responses.

Before data collection, informed consent was obtained from each participant. The consent process was conducted verbally in Somali, with the enumerators ensuring that all participants understood the purpose of the study, the voluntary nature of their participation, and their right to withdraw at any time without penalty. Participation was voluntary, and there were no incentives provided to encourage participation. Participants were informed that their responses would remain confidential and that they could refuse to answer any question without consequence.

The completed questionnaires were securely stored in a password-protected database. Data were anonymized by assigning unique identification numbers to each participant, ensuring that no personally identifiable information was linked to the responses. In cases where there were missing data, enumerators attempted to follow up with the participants to fill in the missing information, especially for critical questions. If follow-up was not possible or if data remained incomplete, the missing data were marked as "not answered" in the database. The analysis was performed with an understanding that some responses might be incomplete, and appropriate statistical techniques for handling missing data were applied.

### Data analysis

#### Quantitative data analysis

Quantitative data were checked for completeness, consistency, and cleaned before analysis. The cleaned data were entered into SPSS version 27 for statistical analysis. Descriptive statistics (frequencies, percentages, and summary measures) were used to describe participants’ socio-demographic, obstetric, healthcare provider, and facility-related characteristics. Bivariate logistic regression was performed to identify candidate variables associated with respectful maternity care (RMC). Variables with a p-value < 0.25 were entered into multivariable logistic regression analysis. In the final model, variables with p-value < 0.05 were considered statistically significant. Adjusted odds ratios (AOR) with 95% confidence intervals (CI) were used to report the strength of associations. Multicollinearity was assessed using the Variance Inflation Factor (VIF), and all values were below 4. Model fitness was checked using the Hosmer–Lemeshow goodness-of-fit test (*p* = 0.660).

#### Qualitative data analysis

Qualitative data were audio-recorded, transcribed verbatim in Somali, and translated into English. The data were then analyzed manually using Braun and Clarke’s six-step thematic analysis approach**,** supported by Open Code software version 4.02 for coding organization.

The steps included:

1. Familiarization with the data through repeated reading of transcripts
2. Generation of initial codes from meaningful statements
3. Searching for patterns and grouping codes into potential themes
4. Reviewing and refining themes for consistency
5. Defining and naming final themes
6. Producing the final narrative report with illustrative quotes

The analysis focused on identifying barriers and facilitators of respectful maternity care from participants’ perspectives.

#### Data quality assurance

For quantitative data, the questionnaire was initially prepared in English and translated into Somali and then translated back into English by language experts for consistency check. All data collectors received two days of training focusing on the data collection tools, proper administration of the questionnaire, and ethical considerations. The questionnaire was pre-tested with 10% of the sample size (43 women) in Bokolmayo refugee camp (one of the five Melkadida refugee camps), which was excluded from the main study. Ongoing supervision and monitoring were conducted by principal investigator throughout data collection process. Questionnaires were reviewed for accuracy and completeness before, during, and after data collection.

## Trustworthiness of qualitative data

To ensure trustworthiness, the following strategies were applied:

- Credibility: Participants were purposively selected based on experience with maternity care. Prolonged engagement and repeated probing were used during interviews to ensure depth of information. Member checking was conducted by summarizing key points during interviews for confirmation.
- Dependability: A clear audit trail was maintained, including documentation of interview guides, coding steps, and theme development procedures. The same interview guide was used across participants to ensure consistency.
- Confirmability: Audio recordings, verbatim transcripts, and field notes were preserved to ensure findings reflected participants’ actual responses. Reflexive notes were maintained to reduce researcher bias.
- Transferability: Thick description of the study setting, participants, and context was provided to allow readers to judge applicability to similar refugee or humanitarian settings.

### Reflexivity

The research team maintained reflexivity throughout the study by acknowledging how researchers' backgrounds could influence data collection and interpretation. The principal investigator, a Somali-speaking health professional with experience in refugee settings, conducted or supervised the interviews. While his linguistic and cultural familiarity facilitated rapport and contextual understanding, his perceived affiliation with the health system could have influenced participants' responses. To minimize this, participants were informed that participation was voluntary, confidential, and unrelated to healthcare or humanitarian assistance, and data collectors were independent of the study health centers. Interviews were conducted in Somali, translated into English, and verified by a bilingual team member to preserve meaning. Coding and interpretation were cross-checked among multiple authors (KB, AN, and ZGA) to enhance credibility and reduce individual bias, although some social desirability and interpretive bias cannot be entirely excluded.

## Results

### Socio-demographic characteristics of respondents

A total of 417 participants were included in the study, yielding a response rate of 98.6%. The largest proportions of respondents were aged 20–24 years (32.4%). Most were married (93.5%) and resided within the refugee camps (97.8%). More than half had no formal education (55.4%) and 72.9% were housewives. In terms of income, 58.5% reported a monthly household income of less than ETB 3000 (Table 1).

**Table 1:** Presents the detailed socio-demographic characteristics of respondents

Types of variables	Category	Frequency	Percent
Age	15–19	90	21.6
	20–24	135	32.4
	25–29	80	19.2
	≥ 30	112	26.8
Residence	In camp	408	97.8
	Out of the camp	9	2.2
Marital status	Married	390	93.5
	Divorce	24	5.8
	Widowed	3	0.7
Education status	No formal education	231	55.4
	Primary	92	22.1
	Secondary and above	94	22.5
Mother’s occupation	Salaried Employment NGO/GO	36	8.6
	Merchant/Private business	77	18.5
	House wife	304	72.9
Household monthly income	< ETB3000	244	58.5
	≥ ETB3000	173	41.5

### Obstetric characteristics of respondents

Regarding obstetric history, 42.7% of women were grand multiparous (≥ 4 births), and 39.1% had delivered three times at a health facility. About 69.3% had antenatal care (ANC) follow-up in their most recent pregnancy; however, only 38.6% attended four or more ANC visits. The majority delivered via spontaneous vaginal delivery (87.3%) and 60.7% gave birth at night. Most women (80.1%) reported no complications, and 96.9% had a live birth outcome. Nearly all participants (97.8%) were accompanied during labor, and 55.2% stayed less than 12 h after delivery (Table 2).

**Table 2:** Presents the obstetric characteristics of respondents

Types of variables	Category	Frequency	Percent
Parity	One	26	6.2
	Two	61	14.6
	Three	152	36.5
	Four and more	178	42.7
Number facility delivery	One	44	10.6
	Two	116	27.8
	Three	163	39.1
	Four and more	94	22.5
Maternal ANC follow up	Yes	289	69.3
	No	128	30.7
Number of ANC visits	< 4	256	61.4
	≥ 4	161	38.6
Mode of recent delivery	SVD/Normal	364	87.3
	Assisted/episiotomy	48	11.5
	Instrumental delivery	5	1.2
Time of the delivery	Day	164	39.3
	Night	253	60.7
Complication during delivery	Maternal	62	14.9
	Neonatal	10	2.4
	Both	11	2.6
	No	334	80.1
Outcome of the delivery	Live	404	96.9
	Death	13	3.1
Companion during delivery	Yes	408	97.8
	No	9	2.2
Hours stayed after delivery	< 12 h	230	55.2
	≥ 12 h	187	44.8

### Healthcare provider characteristics

Most deliveries (88%) were attended by midwives, and 68.1% of providers were female. In addition, 67.9% of deliveries were attended by a single healthcare provider (Table 3).

**Table 3:** Summarizes healthcare provider characteristics

Types of variables	Category	Frequency	Percent
Profession of HCPs	Doctor	22	5.3
	Midwives	367	88
	Nurse/HO	13	3.1
	Unskilled/TBA	15	3.6
Number of HCPs	One	283	67.9
	Two or more	134	32.1
Sex of HCPs	Male	133	31.9
	Female	284	68.1

### Health facility infrastructure

The majority of respondents (97.1%) reported the availability of adequate infrastructure such as waiting rooms, delivery rooms, and beds. More than half reported a sustained electricity supply (63.8%), availability of clean water (51.1%), and hygiene facilities (56.1%). Slightly over half (54.9%) perceived the facility as clean and well maintained (Table 4).

**Table 4:** Presents health facility infrastructure characteristics

Type of variable	Category	Frequency	Percent
Waiting rooms, separated delivery rooms, and enough beds	Yes	405	97.1
	No	12	2.9
Sustained supply of electricity	Yes	266	63.8
	No	151	36.2
Clean drinking	Yes	213	51.1
	No	204	48.9
Hygiene facilities	Yes	234	56.1
	No	183	43.9
Facility cleanliness and maintenance	Yes	229	54.9
	No	188	45.1

### Status of respectful maternity care

**Fig. 1:**
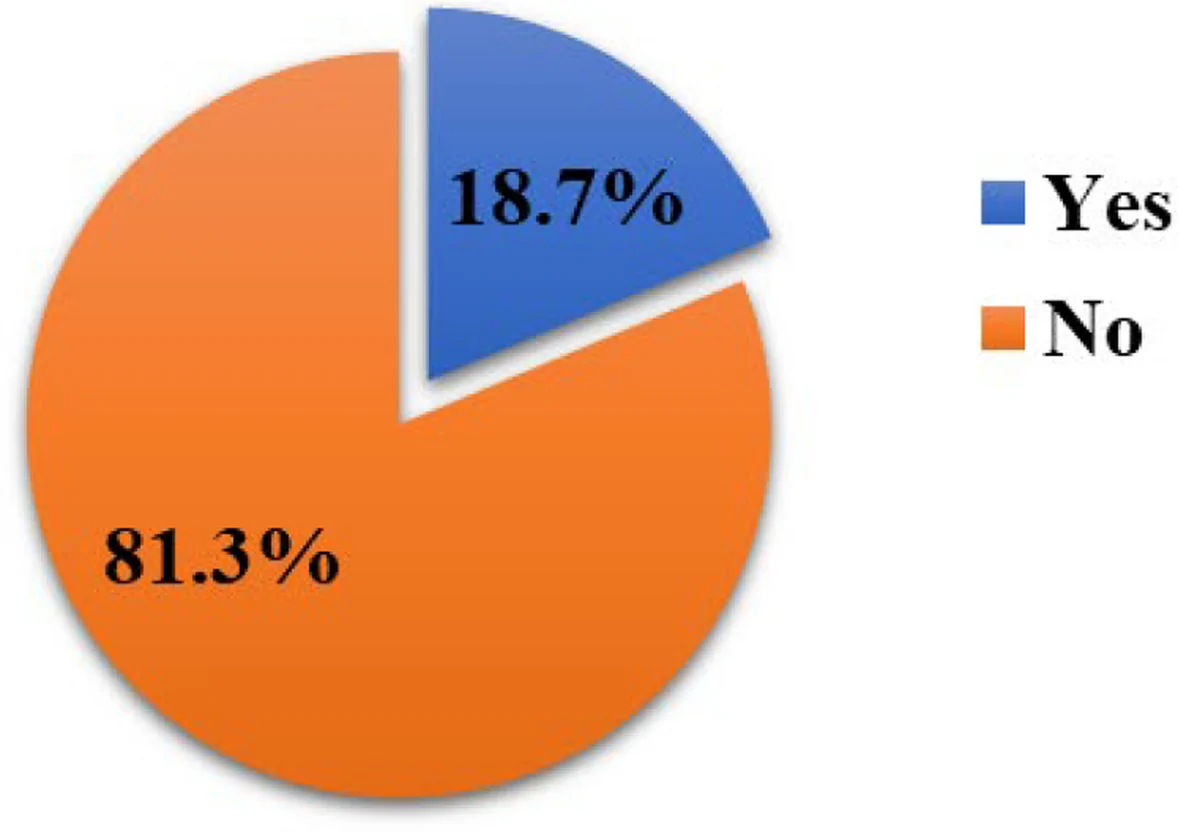
Presents the status of respectful maternity care

Overall, only 18.7% of women received respectful maternity care (RMC), while 81.3% reported experiencing disrespect or abuse during facility-based childbirth (Fig. 1).

### Categories of respectful maternity care

**Fig. 2:**
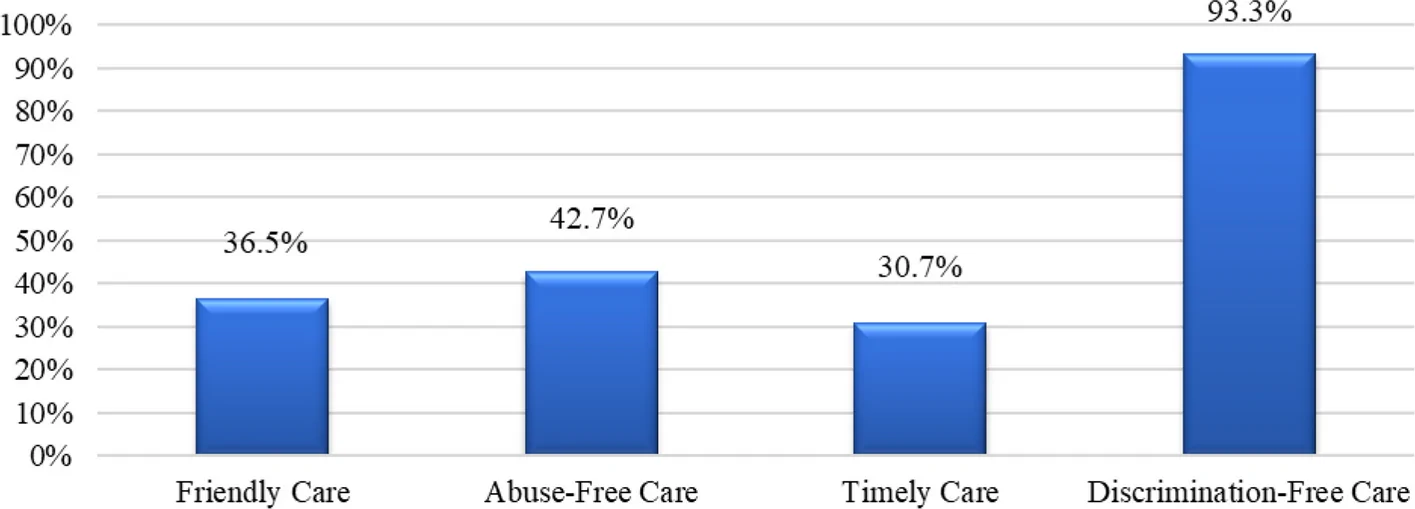
Illustrates RMC by category**.** NB: Each category was measured independently and each percentage represents the proportion of the respondents who answered yes to all items in that specific category

Among the four domains of RMC, 36.5% reported friendly care, 42.7% experienced abuse-free care, 93.3% reported discrimination-free care, and only 30.7% received timely care (Fig. 2).

### Items of respectful maternity care

RMC was assessed using 15 items across four domains. In friendly care, 89.2% reported being treated in understandable language. In abuse-free care, 93.8% reported not being slapped during delivery. In timely care, 85.4% were allowed to practice cultural rituals. In discrimination-free care, 94.7% reported not being insulted due to personal attributes (Table 5).

**Table 5:** Presents detailed item-level responses for RMC

Category of RMC	Items of RMC under each category	Experienced RMC			
		Yes	%	No	%
Friendly care	Cared with a kind approach	331	79.4	86	20.6
	Cared in a friendly manner	308	73.9	109	26.1
	Cared with positive talk about pain and relief	249	59.7	168	40.3
	Cared with showing concern and empathy	296	71	121	29
	Cared with respect as an individual	277	66.4	140	33.6
	Cared with level and understandable language	372	89.2	45	10.8
	Cared with calling by my name	217	52	200	48
Abuse-free care	HWs responded to my needs (whether asked or not)	197	47.2	220	52.8
	HWs slapped me during delivery for any reason®	391	93.8	26	6.2
	HWs shouted at me for not following instructions®	356	85.4	61	14.6
Timely care	Kept waiting long before receiving service®	190	45.6	227	54.4
	I was allowed to practice cultural rituals in the facility	356	85.4	61	14.6
	Service provision was delayed because of the health facility’s internal problem ®	234	56.1	183	43.9
Discrimination-free care	HWs treated me poorly due to personal attributes®	394	94.5	23	5.5
	HWs insulted me or my companions due to personal attributes®	395	94.7	22	5.3

® Reversed

### Multivariable logistic regression analysis of respectful maternity care

In bivariate analysis, twelve variables including age, marital status, monthly household income, number of previous facility deliveries, number of ANC visits, mode of delivery, time of delivery, complications during delivery, length of post-delivery stay, number of healthcare providers (HCPs), sex of HCPs, and facility infrastructure—were associated with respectful maternity care (RMC) at p-value < 0.25. These variables were included in the multivariable logistic regression model based on both statistical significance and theoretical relevance.

After adjusting for potential confounders, several factors remained significantly associated with experiencing RMC (*p* < 0.05). Women aged 25–29 years had higher odds of experiencing RMC compared to the reference group of women aged 15–24 years [AOR = 3.458, 95% CI: 1.547–7.730]. Women who attended four or more ANC visits had higher odds of experiencing RMC compared to those with fewer than four visits [AOR = 3.765, 95% CI: 1.844–7.687]. Daytime deliveries were associated with higher odds of RMC than nighttime deliveries [AOR = 2.203, 95% CI: 1.134–4.280]. Women who stayed at the facility for less than 12 h after delivery had higher odds of receiving RMC compared to those who stayed 12 h or longer [AOR = 3.819, 95% CI: 1.783–8.178]. Deliveries attended by male HCPs were associated with higher odds of RMC compared to those attended by female HCPs [AOR = 3.197, 95% CI: 1.586–6.446]. Finally, facility infrastructure was also associated with RMC: women delivering in facilities with moderate infrastructure had lower odds [AOR = 0.397, 95% CI: 0.178–0.889], and those in facilities with poor infrastructure had substantially lower odds [AOR = 0.090, 95% CI: 0.033–0.245], compared to women in facilities with good infrastructure.

These findings indicate associations rather than causal relationships, highlighting factors that may influence the likelihood of experiencing RMC among Somali refugee women in Melkadida refugee health centers. Reference categories for each variable are clearly indicated in (Table 6).

**Table 6:** Multivariable logistic regression analysis of respectful maternity care among Somali refugee women during facility-based childbirth in Melkadida refugee health centers, Ethiopia, March 2025

Variable	Category	Experienced RMC		COR [95% CI]	AOR [95% CI]
		Yes	No		
Age	15–24	32(14.2%)	193(85.8%)	1	
	25–29	27(33.8%)	53(66.2%)	3.073 (1.694, 5.574) **	3.458 (1.547, 7.730) *
	≥ 30	19(17%)	93(83%)	1.232 (0.663, 2.289)	1.494 (0.691, 3.229)
Marital status	Married	77(19.7%)	313(80.3%)	1	
	Not in marital union	1(3.7%)	26(96.3%)	0.156 (0.021, 1.170)	0.119 (0.014, 1.030)
Monthly HH income	≥ ETB 3000	45(26%)	128(74%)	2.248 (1.363, 3.706) *	0.810 (0.418, 1.570)
	< ETB 3000	33(13.5%)	211(86.5%)	1	
Previous facility delivery	1	12(27.3%)	32(72.7%)	1	
	2	18(15.5%)	98(84.5%)	1.633(0.784, 3.401)	2.221 (0.756, 6.525)
	≥ 3	48(18.7%)	209(81.3%)	0.800 (0.442, 1.446)	0.995 (0.458, 2.162)
Number of ANC visits	≥ 4	17(10.6%)	144(89.4%)	2.650 (1.485, 4.728) *	3.765 (1.844, 7.687) **
	< 4	61(23.8%)	195(76.2%)	1	
Recent mode of delivery	SVD	74(20.3%)	290(79.7%)	3.126 (1.093, 8.939) *	2.557 (0.536, 12.196)
	Assisted	4(7.5%)	49(92.5%)	1	
Time of delivery	Day	52(31.7%)	112(68.3%)	4.054 (2.404, 6.834) **	2.203 (1.134, 4.280) **
	Night	26(10.3%)	227(89.7%)	1	
Complication during delivery	Yes	5(6%)	78(94%)	1	
	No	73(21.8%)	261(78.2%)	4.363 (1.703, 11.177) *	2.513 (0.654, 9.654)
Length of post-delivery stay at the facility	≥ 12 h	13(6.9%)	174(93.1%)	1	
	< 12 h	65(28.3%)	165(71.7%)	5.273 (2.801, 9.924) **	3.819 (1.783, 8.178) **
Number of HCPs	1	47(16.6%)	236(83.4%)	1	
	≥ 2	31(23.1%)	103(76.9%)	1.511 (0.908, 2.515)	1.832 (0.910, 3.691)
Sex of HCPs	Male	47(35.3%)	86(64.7%)	4.460 (2.664, 7.467) **	3.197 (1.586, 6.446) *
	Female	31(10.9%)	253(89.1%)	1	
Facility infrastructure	Good	57(34.8%)	107(65.2%)	1	
	Moderate	15(16.9%)	74(83.1%)	0.381 (0.200, 0.723) *	0.397(0.178, 0.889) *
	Poor	6(3.7%)	158(96.3%)	0.071 (0.030, 0.171) **	0.090 (0.033, 0.245) **

NB: * = statistically significant at *p* value < 0.05, ** = statistically significant at *p* value < 0.01, 1 = reference category, *COR* Crude Odds Ratio and *AOR* Adjusted Odds Ratio

*HCPs* healthcare providers, *HH* household, *SVD* spontaneously vaginal delivery, *ETB* Ethiopian Birr, *ANC* antenatal care, not in marital union = (divorced, widowed)

### Qualitative results

A total of 16 participants were recruited for the qualitative study, including six women, five healthcare providers, and five community leaders, to capture diverse perspectives on respectful maternity care. The study primarily focuses on assessing respectful maternity care (RMC) among Somali refugee women, with emphasis on their lived experiences. However, healthcare providers and community leaders were included in the qualitative component to provide system-level and socio-cultural perspectives influencing the provision and utilization of RMC. Their inclusion enabled triangulation of findings and a more comprehensive understanding by contextualizing women’s experiences within broader health system and community dynamics.

The mean age of participants was 36.2 years (± 14.6). Interviews were audio-recorded, translated, and transcribed verbatim, and the data were systematically coded using Open Code software (version 4.02). Thematic analysis was conducted iteratively, resulting in the identification of three main themes and eight corresponding subthemes, which are presented in Table 7. Measures such as peer debriefing and review of coded transcripts were applied to ensure the credibility and trustworthiness of the qualitative findings.

**Table 7:** Main themes and sub-themes emerged through in-depth interview with HCPs, community leaders, and women on RMC and associated factors in Melkadida refugee camps, Ethiopia, 2025 (*n* = 16)

Main themes	Sub-themes
Healthcare provider-related barriers	Lack of training and knowledge on RMC
	Staff shortage and high workload
	Less experienced provider
Perceived disrespect and interpersonal barriers	Lack of compassion
	Poor communication and deprioritizing emotional needs
Health facility-related factors	Poor facility infrastructure
	Shortage of medications and supplies
	Lack of accountability and weak facility management

### Main theme: Barriers related to healthcare providers

Barriers related to healthcare providers make it difficult to provide RMC to women during facility-based childbirth. These issues often stem from unfavorable working conditions, such as lack of training, staff shortages, and heavy workloads, which affect their performance, job satisfaction, and ability to treat patients with respect and care.

### Subtheme: Lack of training and knowledge gap

Most HCPs have not received proper training in RMC, leaving them unprepared to meet the emotional and communicational needs of mothers.

“*I do not know what RMC means. We have not received any training on RMC. We haven’t been informed of any specific standards. We simply do what we can.”* (HCP3, female 21 years).

### Subtheme: staff shortage

Heavy patient flow makes it difficult for healthcare providers to deliver timely and attentive care. Staff often faces pressure during peak hours, impacting the timely and quality of care.

*"We do not have enough staff, and the patient flow is high. It is not easy to manage 3 to 4 labor; especially when you are the only one on duty.”* (HCP1, female 23 years).

“*I was in labor and another woman was giving birth at the same time. He (HCP) was so busy helping going back and forth to her and to me. You could see he was doing his best but one person cannot do so much all the care you need, while it’s not his fault you miss by rushing*.” (Women2, 27 years).

### Subtheme: Less experienced provider

Many felt that inexperience among HCPs sometimes made the situation worse. This lack of experience led to hesitation during emergencies, reduced trust in care, and increased anxiety among mothers.

“*Even though many female providers have been recruited, the situation hasn’t improved. In fact, it’s become worse in some ways. These young girls, though female, are inexperienced and not well-trained… not active as male.” (Community Leader2, Male, 45 years).* While such perceptions reflect participants' subjective views in this setting, they may be shaped by the recent influx of younger, less experienced female staff and differences in shift assignments, rather than indicating an inherent superiority of male providers.

### Main theme: Perceived disrespect and interpersonal barriers

Participants reported neglect for their request for help or pain, leaving women feeling uncared for during vulnerable times. These experiences contributed to a deep sense of neglect affecting their overall perception of the care they received. Even when medical issues were positive the emotional impact of being ignored during such critical times led to dissatisfaction and mistrust in the healthcare system.

### Subtheme: Lack of compassion

The participants stated that lack of compassion made women feel uncared and disrespected while they were at one of their most vulnerable times. Such attitudes deterred some women from accessing facility-based childbirth.

*“The TBA mother was the only one who treated me with kindness. She supported me throughout. Other staff ignored me and went to sleep. I think I ought to have received dignity and respect. The baby was born right at the door of the delivery room at the floor. During labor, I didn’t receive any respect and medical attention from staff except that TBA mother”. (Women5, 35 years).*

### Subtheme: Poor communication and deprioritizing

The absence of effective communication between mothers and HCPs is one of the key factors lowering the trust and quality of maternity care. Often, the focus on clinical treatment, which is important for preventing complications, may overlook the emotional and ethical needs of the mother.

*“Sometimes when you are so busy and managing critical cases, it is difficult always to explain to the mother what you are doing such times our main responsibilities are to save the life of mother and baby… immediate medical actions should come first*”. (HCP4, male 26 years).

“*Nobody explained to me what was happening during labor, but I was treated and gave birth safely. What we need is not their words or explanation, but we simply want the appropriate care at the right time”. (women*2, 19 years).

### Main theme: Health facility-related factors

This theme highlights issues within healthcare facilities that hinder the provision of respectful maternity care. Shortage of essential resources, poor facility infrastructure, and lack of accountability and weak facility management also contribute to substandard care.

### Subtheme: Poor facility infrastructure

Poor facility infrastructures and unclean environments were frequently mentioned as barriers to respectful maternity care. These limitations not only made mothers feel uncomfortable but also affected the ability of HCPs to provide proper attention and dignity during facility-based childbirth.

“*Our electricity sometimes cuts off and we have to use our phone flashlights and deliver the baby. Imagine using your phone while you are delivering … we are currently experiencing a water shortage, there is no running water from the main line… and it is not always enough which makes routine cleaning difficult, you may see strains on the floor.”* (HCP5, female 20 years).

### Subtheme: Shortage of medication and supplies

Inadequate essential medications and supplies can have negative effects on the standard of care provided to patients, which may also increase the possibility of mistrust in the health system.

*“There is a big shortage of essential medication, investigations, and other medical supplies. When a mother comes to receive full care, either the prescribed medications/investigations aren’t available or she doesn’t receive complete care. We can clearly explain what we can and cannot provide. But when nothing is available at the facility, some mothers become upset and say things like, ‘If nothing is available, what are you even working for? That’s a real challenge for us’”.* (HCP4, male 26 years).

*“During my pregnancy with this child, I kept visiting the health center every day, hoping to receive the necessary medications and tests. However, each time, they told me to return tomorrow or medication was not available.” (women4, 35 years).*

### Subtheme: Lack of accountability

The absence of monitoring and disciplinary systems contributes to the persistence of abusive treatment toward women. When healthcare providers are not held accountable for their actions, inappropriate behaviors may go unchecked and become normalized over time. Without clear consequences, fosters an environment where such conduct is tolerated or overlooked, ultimately undermining efforts to ensure respectful maternity care.

*“When someone checks in on your work, it helps you improve. Sometimes they correct mistakes, and sometimes they commend you for doing a good job. If you’re criticized, you work on improving it. If you're praised, you're encouraged to keep doing better.”* (HCP4, female 24 years).

*“If staff are not properly attended to or did something wrong with women, no one or responsible persons are held accountable.” (Community leader4, female 41 years).*

#### Convergent mixed-methods findings

Despite high rates of facility-based deliveries among Somali refugee women in the Melkadida refugee camps, only 18.7% of women reported experiencing overall respectful maternity care (RMC). Deficiencies were particularly evident in timely care (30.7%) and friendly care (36.5%), although discrimination-free care remained high (93.3%). Multivariable analysis identified key predictors of RMC, including the sex of healthcare providers and the number of antenatal care (ANC) visits, with women attended by male providers and those who attended four or more ANC visits more likely to experience RMC.

These quantitative findings are contextualized and enriched by qualitative insights. Some women described receiving more attentive and confident care from male providers. These qualitative perceptions help contextualize the quantitative association between provider sex and respectful maternity care but should not be interpreted as evidence that provider gender itself determines the quality of care. Rather, the observed association may reflect underlying contextual factors such provider experience, shift assignments, workload distribution, or other organizational dynamics that were not measured directly in this study. Healthcare providers also noted that frequent ANC visits fostered trust and improved communication, enabling them to better address women’s emotional and informational needs during labor. Qualitative themes also highlighted structural and interpersonal barriers such as provider inexperience, staff shortages, poor communication, and limited facility infrastructure that contributed to gaps in RMC, helping to explain the quantitative associations with post-delivery stay, time of delivery, and facility conditions.

The contrast between the high rate of discrimination-free care and the low overall RMC scores is particularly notable. While overt discrimination was uncommon, qualitative findings show that gaps in timeliness, friendliness, communication, and facility resources created barriers to fully respectful care. This demonstrates that both interpersonal and system-level factors shape women’s experiences. Taken together, the integration of quantitative and qualitative findings illustrates that provider characteristics, engagement through ANC, and structural conditions are all critical to promoting respectful maternity care, while system-level and interpersonal challenges remain important constraints.

## Discussion

This study aimed to assess the status of respectful maternity care (RMC) and associated factors during facility-based childbirth among Somali refugee women who gave birth at health centers in the Melkadida refugee camps, Ethiopia. Beyond describing prevalence, this study contributes context-specific evidence on RMC within a protracted humanitarian setting, where health services operate under structural constraints distinct from routine public health systems. Understanding RMC in refugee contexts is critical, as women in such settings experience compounded vulnerability due to displacement, limited resources, and fragile service delivery systems.

The overall magnitude of RMC in this study was low (18.7%), while a significant proportion (81.3%) of women experienced some form of disrespect and abuse (D&A). This finding is higher than the studies in central India (0% RMC, 100% D&A) [12], in Arba Minch, Ethiopia (1.1%) [31], and in Benishangul Gumuz, Ethiopia (12.6%) [28]. Methodological differences such as study design, sample size, and data collection procedures and instruments may explain these variations. For instance, the Benishangul Gumuz study employed direct observations, which may have provided a more objective and minimized recall and social desirability bias, but it may have affected the Hawthorne effect [28]. In Arba Minch study birth attendants used outdated procedures including the use of fundal pressure to eject babies and suturing of episiotomies without the use of local anesthesia, which significantly compromises and drops women’s status of RMC [31]. The study done in India involved a relatively insufficient sample size with only (*n* = 150) and a consecutive sampling technique, which may limit the representativeness and generalizability of the findings [12].

However, this study was much lower than the findings from Iran Tabriz city (63.42%) [32], Nigeria (69.9%) [13], Southwest Ethiopia (81.2%) [25], Addis Ababa public hospitals, Ethiopia (65.8%) [33], Northwest Amhara referral hospitals, Ethiopia (56.3%) [26], and South Gondar Zone, Ethiopia (39.4%) [34]. The differences in the findings might be due to variations in study settings, participant backgrounds, cultural contexts, and health system capacity. Unlike stable hospital environments, refugee camp facilities often operate with limited infrastructure, inconsistent electricity, heavy workload, and high staff turnover. This suggests that respectful maternity care is not solely dependent on provider attitude but also on structural and system-level determinants.

The low overall prevalence of RMC should be interpreted in the context of the study's strict all-or-none operationalization, which classified women as having received RMC only if they fulfilled all 15 items. Although this validated approach aligns with the human rights principle that every woman is entitled to care free from any form of D&A [1, 7], it may underestimate women's experiences of partial respectful care because a single unmet item resulted in classification as D&A. Thus, the overall estimate represents comprehensive respectful maternity care, while the domain-specific findings offer a more nuanced picture of women’s experiences during childbirth.

Among RMC categories, discrimination-free care was the most commonly experienced, reported by (93.3%) of participants. This finding was higher than studies in southwest Ethiopia (81.9%) [25], Northwest Amhara referral hospitals, Ethiopia (82.4%) [26], and Harar hospitals, Eastern Ethiopia (45.4%) [35]. The higher rates in this study may be due to the greater awareness and sensitivity around the issues towards discrimination in refugee settings where the population is more vulnerable, as well as an organizational working culture that emphasizes inclusiveness and equity in care provision in humanitarian settings. However, despite high discrimination-free care, the overall RMC level remained low. This discrepancy highlights the multidimensional nature of RMC measurement, where strong performance in one domain does not compensate for deficiencies in other domains under strict classification criteria.

Abuse-free care was reported (42.7%). This finding is lower than the study in southwest Ethiopia (77%) [25], Northwest Amhara referral hospitals (62%) [26], Tirunesh Beijing Hospital, Addis Ababa (83.6%) [36], and Harar hospitals, Eastern Ethiopia (45.5%) [35]. These deviations may be due to differences in health system performance and healthcare provider-related factors. In refugee settings, provider workload, limited training refreshers, and constrained supervision mechanisms may further exacerbate interpersonal care challenges.

Friendly care was reported (36.5%) of participants. This result is lower than the study in Tirunesh Beijing Hospital, Addis Ababa (80.9%) [36], and Harar hospitals, Eastern Ethiopia (59.8%) [35]. This may be due to provider attitudes and challenging work conditions in under-resourced refugee settings. Qualitative findings support that communication gaps and emotional exhaustion among staff may reduce empathetic engagement with laboring women.

Timely care at (30.7%). This figure is higher than the study in Bahir Dar (14.4%) [37], but considerably lower than the studies in Harar hospitals, Eastern Ethiopia (76%) [35], Southwest Ethiopia (84.1%) [25], and Northwest Amhara referral hospitals (96.30%) [26]. This deviation may be due to a weak health system and limited accountability in refugee settings. Qualitative insights support this, showing how staffing shortages especially during shift time and heavy workloads hinder the provision of timely and adequate care. This suggests a time-of-delivery inequity in respectful care provision, particularly disadvantaging women delivering at night.

This study found that women aged 25–29 years were more likely to receive RMC than those aged 15–24, consistent with findings from Ilu Ababor and Tanzania [26, 38]. In the refugee context, older women may have greater familiarity with facility processes and stronger communication confidence, potentially influencing their care experience.

Women who had four or more ANC visits were 3.77 times more likely to experience RMC. This finding is consistent with findings from Northwest Amhara Ethiopia and Southwest Ethiopia [25, 26]. This may be because frequent contact builds trust and awareness. However, the qualitative part indicates that frequent ANC visits alone do not always guarantee respected care when systemic resource shortages persist. This underscores that service utilization alone is insufficient without concurrent quality improvement within facilities.

Women who gave birth in the daytime were 2.2 times more likely to experience respectful care compared to nighttime deliveries. This aligns with studies in Nepal [38], and Iran [30]. In humanitarian settings, nighttime services may be particularly vulnerable due to limited staffing coverage and infrastructure instability.

The odds of receiving RMC were 3.2 times higher among women attended by male healthcare providers. This finding is contextually noteworthy given Somali cultural norms that often favor female providers [39], suggesting that perceived provider responsiveness may outweigh gender preference in high-workload environments. However, this association should be interpreted cautiously, as it may reflect differences in provider experience, workload distribution, staffing patterns, or other organizational factors rather than provider gender itself. Qualitative findings suggested that male providers were sometimes perceived as more senior and experienced, which may have influenced women's perceptions of responsiveness and confidence in care.

Shorter post-delivery stays of less than 12 h were 3.8 times more likely to experience RMC. Longer exposure to strained facility environments may increase the likelihood of encountering service gaps or communication breakdowns. Women who delivered in facilities with moderate or poor infrastructure were significantly less likely to receive RMC. This reinforces that respectful maternity care is structurally embedded, and improvements in physical infrastructure, lighting, sanitation, and staffing stability are essential components of dignity-preserving care in humanitarian settings. This finding is consistent with a systematic review conducted in Ethiopia [40].

The intersection of migration, structural inequities, and sexual and reproductive health and rights (SRHR) creates significant barriers to accessing quality maternal healthcare in refugee settings [41, 42]. Refugee women often experience multiple, overlapping disadvantages, including language barriers, limited health literacy, and socio-economic constraints, which can restrict their access to respectful and equitable care [43, 44]. Consistent with these broader perspectives, our findings show that women's experiences of respectful maternity care were shaped not only by provider–patient interactions but also by health-system constraints, including inadequate infrastructure, staffing shortages, unreliable essential services, and heavy workloads. These findings suggest that improving respectful maternity care in refugee settings requires both provider-level interventions and broader health system strengthening to address the structural factors influencing care.

### Strengths of the study

To the best of available evidence, this is the first study to assess RMC among refugee women in Ethiopia. The study employed a mixed-methods design and a nationally validated tool, providing a comprehensive understanding of the current status and contextual barriers. It also incorporated perspectives from women, healthcare providers, and community leaders.

### Limitations of the study

The study relied on self-reported data, which may be subject to recall and social desirability bias, potentially affecting the accuracy of reported experiences. Additionally, the facility-based recruitment strategy, which included only women who returned for postnatal or related services, may introduce selection bias, as women who did not seek follow-up care possibly due to negative experiences or access barriers were not represented. The measurement of respectful maternity care (RMC) used a strict all-or-none classification, where a woman was considered to have received RMC only if all items were reported as “yes.” This approach may have contributed to a lower overall estimate of RMC, as a single negative response classified the entire experience as non-respectful care, and the binary overall indicator should not be read as evidence of uniformly poor care across all dimensions, the domain and item level results provide a more nuanced picture of where care was and was not respectful. Future studies may consider reporting graded or domain-weighted RMC scores alongside the strict composite indicator to better capture partial respectful care. In the qualitative component, women who participated in the quantitative survey were excluded to ensure diverse perspectives; however, this limits the ability to directly link qualitative and quantitative findings, which may affect the integration and interpretation of results. The study also did not include policymakers or representatives from donor agencies, which may limit the breadth of stakeholder perspectives. Finally, the lack of comparable refugee-specific data in Ethiopia constrained contextual comparisons.

## Conclusions

This study assessed the prevalence and associated factors of respectful maternity care (RMC) among Somali refugee women in Melkadida refugee camps, Ethiopia. It found a substantial gap in RMC, with many women reporting disrespect and abuse during childbirth. However, domain level findings show that this gap was concentrated in friendly, abuse-free, and timely care, while discrimination-free care was comparatively high, indicating that care was not uniformly poor across all dimensions. Higher RMC was associated with age 25–29 years, ≥ 4 antenatal care visits, daytime delivery, shorter postnatal stay (< 12 h), and male healthcare providers. Better facility conditions were also linked to improved RMC. Qualitative findings highlighted barriers including poor infrastructure, staff shortages, limited resources, inadequate provider training and attitudes, and poor communication between providers and women.

### Implications for practice

The findings underscore that respectful maternity care (RMC) in this setting is best understood as a health-systems issue rather than solely a matter of individual provider behavior. So, interventions should be prioritized based on this perspective. In refugee settings, reliable electricity and water, adequate supplies, and sufficient staffing are essentials for delivering respectful care. These must be recognized as important humanitarian health-system priorities. At the same time, providers need ongoing training in respectful care practices, particularly given the heightened vulnerability of women in these settings. Policymakers and humanitarian organizations should prioritize both structural and provider-level factors to improve the quality of maternal healthcare in refugee camps. Strengthening accountability mechanisms within health facilities, promoting community awareness, and establishing clear protocols for respectful care could also significantly reduce the occurrence of disrespect and abuse. This study emphasizes that improving respectful maternity care is not solely about life-saving interventions but also about safeguarding the dignity and autonomy of women, particularly in vulnerable settings like refugee camps.

### Recommendations

Policymakers, NGOs, and other relevant stakeholders should strengthen health facility infrastructure, ensure the consistent availability of essential medications, recruit and retain skilled healthcare providers, and implement accountability mechanisms to promote and institutionalize respectful maternity care (RMC). They should also provide regular training for healthcare providers, ensure close supervision during shifts, and reduce unnecessary post-delivery stays where appropriate. Future research should explore why male providers were associated with higher RMC, community-based perspectives and include policymakers to provide a more comprehensive understanding of the issue.

## Data Availability

The datasets generated or analysed during the current study are not publicly available due to the sensitive nature of the data and ethical considerations, but are available from the corresponding author on reasonable request.

## Abbreviations

ANC: Antenatal care
AOR: Adjusted odds ratio
CI: Confidence interval
D&A: Disrespect and abuse
EHSTP: Ethiopian health sector transformation plan
LMICs: Low and middle-income countries
MMR: Maternal mortality ratio
RMC: Respectful maternity care
SBA: Skilled birth attendant
UNHCR: United nations high commissioner for refugees
WHO: World health organization
SSA: Sub-Saharan Africa
SDGs: Sustainable development goals
SPSS: Statistical package for the social science
SRHR: Sexual and reproductive health and rights
